# Molecular Adjuvant Potential of *GCSF* and *MCSF* in Starry Flounder Challenged with *Streptococcus parauberis*

**DOI:** 10.3390/ani15131848

**Published:** 2025-06-23

**Authors:** Min-Young Sohn, Gyoungsik Kang, Kyung-Ho Kim, Ha-Jeong Son, Chan-Il Park

**Affiliations:** 1 Department of Marine Biology & Aquaculture, College of Marine Science, Gyeongsang National University, 455, Tongyeong 650-160, Republic of Korea; thisdancemoment@naver.com (M.-Y.S.); jemma9120@naver.com (H.-J.S.); 2 Department of Aquatic Life Medicine, College of Marine Science, Gyeongsang National University, 455, Tongyeong 650-160, Republic of Korea; gyoungsikkang@gmail.com (G.K.); rsiv94@naver.com (K.-H.K.)

**Keywords:** cytokines, disease resistance, phagocytosis, immune response

## Abstract

In fish, the innate immune system is crucial for rapid defense against pathogens. In this study, we performed transcriptome sequencing using next-generation sequencing (NGS) to identify and characterize granulocyte colony-stimulating factor (*GCSF*) and macrophage colony-stimulating factor (*MCSF*) in starry flounder (*Platichthys stellatus*). The *GCSF* gene (594 bp, 198 aa) features a conserved IL-6 domain, while the *MCSF* gene (621 bp, 207 aa) contains a predicted transmembrane region. Phylogenetic analysis confirmed high evolutionary conservation with other marine species. Quantitative real-time PCR revealed that *GCSF* is highly expressed in the skin, peripheral blood leukocytes, and muscle, with significant up-regulation in immune organs following *Streptococcus parauberis* infection; *MCSF* exhibited a similar tissue-specific expression pattern. Recombinant *GCSF* (*rGCSF*) was produced using a cell-free system and effectively enhanced leukocyte phagocytic activity at an optimal concentration of 150 μg/mL, without causing cytotoxicity in hemolytic assays. In contrast, *rMCSF* exhibited folding issues during purification. These findings highlight the potential of *rGCSF* as a molecular adjuvant to enhance immune responses in aquaculture. This study provides foundational knowledge for developing cytokine-based adjuvants, which could reduce antibiotic dependency and enhance vaccine efficacy in sustainable aquaculture systems.

## Simple Summary

*Granulocyte colony-stimulating factor* (*GCSF*) and *macrophage colony-stimulating factor* (*MCSF*) play key roles in regulating fish immunity, yet their functions in starry flounder remain uncharacterized. Using next-generation sequencing, we identified the starry flounder *GCSF* gene, which harbors a conserved *IL-6* domain, and the *MCSF* gene, which contains a transmembrane region. Both factors show strong evolutionary conservation with other marine species and are highly expressed in the skin, blood leukocytes, muscle, and immune organs following bacterial challenges. Recombinant *GCSF* produced in a cell-free system significantly enhances the phagocytic activity of fish leukocytes without cytotoxicity, indicating its promise as a molecular adjuvant in aquaculture. In contrast, recombinant *MCSF* requires further optimization due to folding issues during purification.

## 1. Introduction

The immune response is a fundamental physiological mechanism by which animals protect themselves from external pathogens and maintain internal homeostasis, playing a crucial role in determining resistance to infections and survival. The immune system is broadly divided into innate and adaptive immunity, which interact to defend the host from infections [1]. Innate immunity serves as the first line of defense, providing a rapid, nonspecific response to invading microorganisms through pathogen-associated molecular pattern (PAMP) recognition receptors and various inflammatory mediators that eliminate pathogens and reinforce early defenses [2]. In contrast, adaptive immunity offers a pathogen-specific defense, forming immunological memory to mount faster and stronger responses upon reinfection. These two systems are complementary, with innate immunity suppressing pathogen spread until adaptive immunity is established [3].

Fish represent an evolutionary model of early immune systems and predominantly rely on nonspecific or innate immune responses. Upon pathogen exposure, fish quickly react through innate immunity to suppress infection. When pathogens persist, adaptive immunity is activated for more sophisticated defense mechanisms [4,5]. However, due to the slower onset of adaptive immunity in fish compared to mammals, innate immunity plays a more critical role [2]. Key components of innate immunity in fish, including lysozymes, acute-phase proteins, and complement proteins, play vital defensive roles and are commonly used as markers of disease resistance [2].

Defense mechanisms against diseases are central to immune responses required for survival in the face of external stimuli or pathogens [6]. These mechanisms involve complex interactions between signaling pathways, cytokines, and growth factors, collectively working to eliminate pathogens and limit infections.

Cytokines act as critical regulators of immune responses, functioning as low-molecular-weight glycoproteins or polypeptides that mediate immune system interactions and coordinate the host defense network [7,8]. Growth factors such as granulocyte colony-stimulating factor (*GCSF*) and macrophage colony-stimulating factor (*MCSF*) are other important cytokines that promote immune cell proliferation and differentiation, playing essential roles in immune responses. *GCSF*, a hematopoietic cytokine, stimulates hematopoiesis in the bone marrow and induces the proliferation and differentiation of key immune cells such as neutrophils. It facilitates the rapid mobilization of immune cells to inflamed tissues, contributing to inflammation resolution [9]. Recent studies in mammals have shown that *GCSF* plays a role in inflammation and resistance to pathogenic infections [10]. Recombinant *GCSF* proteins are widely used as therapeutics for neutropenia in humans [11]. Similarly, *MCSF* promotes macrophage proliferation, enabling these cells to phagocytose and eliminate pathogens in infected tissues.

These immune-related genes have potential as molecular adjuvants. Molecular adjuvants are substances administered alongside vaccines to enhance immune responses [12,13]. In mammals, over 200 cytokines have been identified, many of which enhance humoral and cell-mediated immunity to provide robust protection against infections. Compared to traditional adjuvants like aluminum salts or oil emulsions, cytokines offer advantages by inducing co-stimulatory molecule expression and activating antigen-presenting cells [14,15].

In teleost fish, cytokines similarly orchestrate both innate and adaptive immune responses. Numerous cytokines, including interleukins (e.g., IL-1β, IL-6, and IL-8), tumor necrosis factors (e.g., TNF-α), and colony-stimulating factors (e.g., GCSF and MCSF), have been identified and functionally characterized in various fish species [8]. These cytokines regulate key immune processes such as leukocyte proliferation, phagocytosis, inflammation, and antigen presentation. Importantly, several studies have demonstrated that the use of recombinant cytokines as molecular adjuvants in fish can significantly enhance vaccine-induced antibody production and cellular responses, as observed in olive flounder and rainbow trout [16,17].

Therefore, understanding and harnessing the immunomodulatory functions of fish cytokines, particularly GCSF and MCSF, is vital for developing more effective vaccines and sustainable disease control strategies in aquaculture. These gene-based molecular adjuvants offer a novel approach to enhancing host immunity, reducing antibiotic dependence, and improving disease resistance in economically important aquaculture species like starry flounder.

## 2. Materials and Methods

### 2.1. Ethics Statement and Experimental Animals and Pathogens

All animal experiments were conducted in accordance with relevant Korean regulatory guidelines. The experimental protocol was reviewed and approved by the Institutional Animal Care and Use Committee (IACUC) of the College of Marine Science, Gyeongsang National University (Approval No.: GNU-241104-E0206; Date of Approval: 4 November 2024).

One hundred starry flounder were purchased from a private aquaculture farm in Pohang, Gyeongsangbuk-do, Republic of Korea. The fish were maintained in recirculating seawater systems at 25 ± 1 °C, salinity 30–32 ppt, pH 7.5–7.8, and dissolved oxygen above 6.0 mg/L. During acclimation and prior to infection, the fish were fed a commercial pellet diet (42% crude protein and 8% lipid; Jeil Feed Co., Daejeon, Republic of Korea) at 1.5% of their body weight per day. The experimental fish had an average total length of 21.5 ± 1.3 cm and a mean weight of 128.7 ± 18.2 g.

Before the challenge experiment, three fish were randomly selected and subjected to clinical examination to confirm their health status, after which they were used in the experiment.

The *Streptococcus parauberis* strain PH0710 used in this study was provided and approved for use by the National Institute of Fisheries Science (Busan, Republic of Korea). The strain was identified by sequencing the 16S rRNA gene using the universal primer 27F (5′-AGAGTTTGATCMTGGCTCAG-3′), and sequence homology was confirmed through a BLAST analysis in the NCBI database. A GenBank accession number for this strain is not available. The bacteria were cultured in brain heart infusion (BHI) broth at 27 °C for 24 h, harvested by centrifugation at 3000× *g* for 10 min, and washed twice with sterile PBS. The final bacterial concentration was adjusted to 1 × 10^7^ CFU/mL based on OD_600_ readings and plate counting.

### 2.2. Sequence Validation and mRNA Expression Analysis of GCSF and MCSF in Starry Flounder

#### 2.2.1. Sequence Validation of GCSF and MCSF

To confirm the accuracy of key gene sequences identified by next-generation sequencing (NGS), TA cloning followed by Sanger sequencing was employed. Gene-specific primers were designed targeting the 5′ and 3′ ends of the open reading frame (ORF) regions obtained from NGS data. PCR amplification was performed using ExPrime Taq Premix (2×) (Genetbio, Daejeon, Republic of Korea) in a 20 µL reaction containing 10 µL of premix, 1 µL each of forward and reverse primers, 1 µL of cDNA (synthesized as described in Section 1), and 7 µL of distilled water. The PCR products were separated on a 1.2% agarose gel containing 0.01% SafeView Classic dye (ABM, San Francisco, CA, USA), and target bands were verified using a DNA ladder. The target bands were excised and purified using the QIAquick Gel Extraction Kit (Qiagen, Hilden, Germany) following the manufacturer’s protocol.

For cloning, the purified PCR fragments were ligated into the pGEM-T Easy vector (Promega, Madison, WI, USA) using a standard ligation reaction mixture consisting of 1 µL vector, 5 µL 2X Rapid Ligation Buffer, 1 µL T4 DNA Ligase, and 3 µL PCR product, incubated at 4 °C overnight. The ligation products were introduced into *Escherichia coli* JM109 competent cells by heat-shock transformation. The mixture was incubated on ice for 30 min, heat-shocked at 42 °C for 1 min, and chilled again on ice for 7 min. The transformed cells were recovered in 800 µL of SOC medium at 37 °C for 4 h, followed by centrifugation at 1000× *g* for 10 min.

Transformed cells were plated on LB agar supplemented with ampicillin (Sigma-Aldrich, Saint Louis, MO, USA), X-gal (Thermo Fisher Scientific, Waltham, MA, USA), and IPTG (Generay Biotech, Shanghai, China), and incubated at 37 °C for 12 h for blue-white colony screening. Positive colonies were selected and cultured in LB broth under shaking conditions at 37 °C for 12 h. Plasmid DNA was extracted using the Hybrid-Q™ Plasmid Rapidprep Kit (GeneAll, Seoul, Republic of Korea) according to the manufacturer’s instructions. The resulting plasmids were submitted to Bioneer (Daejeon, Republic of Korea) for Sanger sequencing.

#### 2.2.2. Multiple Sequence Alignment and Phylogenetic Analysis

The confirmed gene sequences were further analyzed to assess sequence similarity and predict functional characteristics through multiple sequence alignment and phylogenetic inference. cDNA sequences were translated into corresponding amino acid sequences using the GENETYX software (version 8.0; SDC Software Development, Japan). Homologous protein sequences were identified using the Basic Local Alignment Search Tool (BLAST) against the National Center for Biotechnology Information (NCBI) database (http://www.ncbi.nlm.nih.gov/blast (accessed on 15 June 2025)).

Functional domains and conserved motifs within the deduced protein sequences were annotated using two databases: PROSITE (http://www.expasy.org/tools/scanprosite/ (accessed on 15 June 2025)) and the SMART (Simple Modular Architecture Research Tool) platform (http://smart.embl-heidelberg.de/ (accessed on 15 June 2025)).

The multiple sequence alignments were performed using ClustalX2 (version 2.1), and the alignment outputs were visualized and edited with the GeneDoc (version 2.7), software. Phylogenetic relationships were evaluated by constructing trees using the neighbor-joining (NJ) method implemented in the MEGA software (version 4.0) based on ClustalW-aligned sequences. The robustness of the phylogenetic groupings was assessed through 1000 bootstrap replicates.

### 2.3. Gene Expression Analysis

The expression levels of key genes were analyzed in healthy and artificially infected starry flounder. Total RNA was extracted from the dissected tissues, converted into cDNA, and analyzed using real-time PCR to measure mRNA expression levels.

#### 2.3.1. Extraction of Total RNA and cDNA Synthesis from Major Tissues of Healthy and Artificially Infected Starry Flounder

To assess the mRNA expression levels of key genes in the tissues of healthy starry flounder, 12 major tissues (the brain, eye, gill, head kidney, heart, intestine, liver, muscle, skin, spleen, stomach, and trunk kidney) were aseptically dissected from five individuals and stored at −80 °C until total RNA extraction. Blood was collected from the caudal vein using 1 mL syringes with 26-gauge needles, pre-treated with heparin sodium (Sigma-Aldrich, St. Louis, MO, USA). Peripheral blood leukocytes (PBLs) and red blood cells (RBCs) were separated using a 53% Percoll solution and centrifugation. The isolated blood samples were immediately used for total RNA extraction and cDNA synthesis. The same procedure was followed for total RNA extraction and cDNA synthesis from tissue samples stored at −80 °C.

To evaluate the time-dependent mRNA expression levels in major tissues following artificial infection, S. parauberis PH0710 was diluted in PBS buffer to a concentration of 1 × 10^3^ CFU per 100 µL, and 100 µL of the bacterial suspension was administered via subcutaneous injection to each healthy starry flounder maintained at 25 ± 1 °C.

*S. parauberis* strain PH0710 was cultured in 15 mL of BHI broth (BD Difco™, Franklin Lakes, NJ, USA) at 27 °C for 24 h with shaking at 200 rpm. The optical density was measured at 595 nm (OD_595_) and adjusted to 0.15, corresponding to approximately 1 × 10^7^ CFU/mL, as confirmed by plate counting. The bacterial suspension was then washed twice with sterile PBS and diluted to a final concentration of 1 × 10^3^ CFU per 100 µL for injection. Five fish were randomly selected at 0, 1, and 12 h, as well as on days 1, 3, 5, and 7 post-infection. The brain, gill, heart, head kidney, liver, intestine, and spleen were dissected and stored at −80 °C until total RNA extraction. The 0 hpi samples were collected immediately after the subcutaneous injection of Streptococcus parauberis. These samples served as internal baseline controls for monitoring time-dependent gene expression changes in response to infection. Although not naïve controls, this approach is commonly used in fish immunology to capture the earliest post-infection state before immune activation begins.

Total RNA was extracted by adding 1 mL of RNAiso Plus solution to the dissected tissue, followed by thorough homogenization using a homogenizer. Next, 100 µL of chloroform (Daejung, Siheung-si, Republic of Korea) was added, and the mixture was vortexed and centrifuged at 13,000 rpm for 10 min at 4 °C. The aqueous phase was carefully transferred to a fresh microcentrifuge tube, and 200 µL of PCI solution (Phenol/chloroform/Isoamyl Alcohol, 25:24:1; Biosesang, Yongin-si, Republic of Korea) was added. The mixture was thoroughly vortexed and centrifuged under the same conditions as previously described. The upper phase was then transferred to a new tube, followed by the addition of 3 µL of recombinant DNase I (5 U/µL; Takara, Kusatsu, Japan) and 20 µL of 10× DNase I buffer to eliminate residual genomic DNA. The reaction was incubated at 37 °C for 30 min.

To remove the enzyme and any remaining contaminants, an equal volume of PCI solution was again added to the reaction, vortexed, and centrifuged at 13,000 rpm for 10 min at 4 °C. The resulting supernatant was combined with 500 µL of isopropanol (Daejung, Republic of Korea), 50 µL of 3 M sodium acetate (Takara, Japan), and 5 µL of Dr. Gen reagent (Takara, Japan), and centrifuged under identical conditions to precipitate the RNA.

Following centrifugation, the supernatant was carefully discarded, and the RNA pellet was washed with 600 µL of 75% ethanol prepared with diethylpyrocarbonate (DEPC)-treated water (Bioneer, Republic of Korea). The sample was centrifuged again at 13,000 rpm for 5 min at 4 °C. After complete removal of the ethanol, the pellet was air-dried briefly and resuspended in 20 µL of DEPC-treated water. The extracted total RNA samples were reverse-transcribed into cDNA using the PrimeScript™ 1st Strand cDNA Synthesis Kit (Takara, Japan) following the manufacturer’s protocol. For cDNA synthesis, 8 µL of total RNA, 1 µL of random primer, and 1 µL of dNTP mixture were combined and incubated at 65 °C for 5 min, followed by 5 min on ice. To initiate cDNA synthesis, 4 µL of 5× PrimeScript buffer, 0.5 µL of RNase inhibitor, 1 µL of PrimeScript reverse transcriptase (RTase), and 4.5 µL of RNase-free distilled water were added to the reaction mixture. The reaction was carried out under the following thermal conditions: incubation at 30 °C for 10 min for primer annealing, followed by 60 min at 42 °C for reverse transcription. The reaction was terminated by heating at 95 °C for 5 min to inactivate the enzyme and stabilize the cDNA.

#### 2.3.2. Measurement of mRNA Expression

The mRNA expression levels of the target genes were measured using quantitative real-time PCR (RT-qPCR) with the SYBR Green method, employing the TB Green™ Premix Ex Taq™ (Tli RNaseH Plus) (Takara, Japan). The PCR reaction mixture contained 12.5 µL of TB Green Premix Ex Taq (2×), 1 µL each of forward primer (10 pmol) and reverse primer (10 pmol), and 1 µL of cDNA template, with sterile distilled water added to a final volume of 25 µL. The sequences and details of the primer sets used are provided in Table 1.

The PCR reactions were performed on the Thermal Cycler Dice^®^ Real Time System III (Takara, Japan) under the following conditions: initial reaction at 50 °C for 4 min, followed by denaturation at 95 °C for 10 min, and 45 cycles of 95 °C for 15 s and 60 °C for 30 s. The cycle threshold (Ct) values were normalized to the Ct values of the EF-1α gene in the starry flounder and quantified using the 2^−ΔΔCt^ method, as described in previous studies [18]. The EF-1α gene was used as the internal reference for normalization due to its validated expression stability across various tissues and experimental conditions in fish. Previous studies have demonstrated that EF-1α is a reliable housekeeping gene for qPCR analyses under immune stimulation and stress conditions in teleosts, including Atlantic cod (*Gadus morhua*) and olive flounder (*Paralichthys olivaceus*) [19,20].

### 2.4. Production of Recombinant Proteins from GCSF and MCSF Genes in Starry Flounder

Recombinant *GCSF* protein and recombinant *MCSF* protein were synthesized using a cell-free protein expression system and the ExiProgen Protein Synthesis System (Bioneer, Republic of Korea) according to the manufacturer’s instructions. Briefly, the respective gene encoding each protein was inserted into the pBIVT-2 vector, which includes a 6×histidine protein fusion partner. The expression was performed at 30 °C in a reaction mixture containing *E. coli* extract with T7 RNA polymerase, ribosomes, and tRNAs necessary for protein synthesis. Target proteins were expressed by the transcription of the DNA sequence into mRNA, followed by the translation of mRNA into proteins using amino acids and an energy source.

The expressed proteins were purified using Ni-affinity chromatography and dissolved in a storage buffer containing 50 mM Tris-Cl (pH 7.6), 100 mM NaCl, 1 mM DTT, 0.1 mM EDTA, 0.05% NaN_3_, and 50% glycerol. The final products were analyzed for specificity and purity using 12% SDS-PAGE. Protein concentrations were measured using the Bradford dye-binding method with the Bio-Rad Protein Assay Kit (Bio-Rad, Hercules, CA, USA), following the manufacturer’s protocol.

### 2.5. Biological Activity Assessment of Molecular Adjuvants

#### 2.5.1. Phagocytic Activity Assessment of Molecular Adjuvants

To evaluate the phagocytic activity of the *rGCSF* protein, leukocytes were isolated from the starry flounder using a 53% Percoll solution. To prepare the 53% Percoll solution, 26.5 mL of Percoll solution was mixed with 2.95 mL of 10× PBS buffer and 20.55 mL of PBS buffer. The solution was distributed into 15 mL Corning tubes (BD Biosciences, Franklin Lakes, NJ, USA) in 4 mL aliquots. The kidney, spleen, and gill tissues were placed on 100 µm cell strainers (BD Biosciences, USA) and carefully homogenized with 2 mL RPMI medium (Sigma-Aldrich, USA). The resulting cell suspension was gently layered on top of the 53% Percoll solution (5 mL per tube) and centrifuged at 500× *g* for 15 min at 20 °C. The purity of the isolated leukocytes was evaluated using flow cytometry. Forward and side scatter gating was used to identify and isolate the leukocyte population, and non-target cells such as debris or erythrocytes were excluded. The gating strategy and representative results are presented in Figure 8. The leukocyte layer was collected into 50 mL Corning tubes (BD Biosciences, USA) and washed with 40 mL PBS buffer by centrifugation at 400× *g* for 10 min at room temperature.

The leukocyte pellet was resuspended in 1 mL Red Blood Cell Lysing Buffer Hybri-Max solution (Sigma-Aldrich, USA) and incubated for 1 min at room temperature to lyse red blood cells. The mixture was then washed with 40 mL PBS buffer and centrifuged again at 400× *g* for 10 min to isolate leukocytes, followed by an additional PBS buffer wash. The isolated leukocytes were used in the subsequent experiments.

Fluorescently labeled *S. parauberis* PH0710 was used as the target microorganism. The pathogen was labeled with fluorescein isothiocyanate (FITC; 10 mg/mL in DMSO). The leukocytes from the starry flounder were prepared at a concentration of 1 × 10^6^ cells/mL. The control group consisted of leukocytes treated with PBS and FITC-labeled *S. parauberis* PH0710, while the experimental groups were treated with *rGCSF* protein at concentrations of 50, 100, 150, and 200 µg/mL, followed by the addition of FITC-labeled *S. parauberis* PH0710. All the samples were incubated at room temperature in the dark for 30 min.

#### 2.5.2. Hemolytic Activity Assessment of Molecular Adjuvants

To assess the safety of *rGCSF*, a hemolytic activity assay was conducted using starry flounder erythrocytes. This assay was designed to evaluate whether *rGCSF* induces cytotoxic effects, as adjuvant candidates must be non-toxic to host cells while enhancing immune responses.

Erythrocytes were isolated from the peripheral blood of the starry flounder using Percoll solution and centrifugation as described in Section 2.5.1. The erythrocytes were suspended in PBS buffer at a concentration of 4%.

*rGCSF* protein was diluted in PBS buffer to prepare six concentration groups: 1, 10, 50, 100, 150, and 200 µg/mL. Each well of a 96-well plate was filled with 50 µL of the 4% erythrocyte suspension and 50 µL of the respective protein suspensions. The plates were incubated at room temperature for 30 min. The positive control group was treated with an equivalent volume of 0.1% Triton X-100 solution (Sigma-Aldrich, USA), while the negative control group was treated with PBS buffer only. After incubation, the plates were centrifuged at 1000× *g* for 10 min. The supernatant was transferred to a new 96-well plate, and absorbance was measured at 540 nm to assess hemolysis.

### 2.6. Statistical Analysis

All the experiments were conducted in triplicate, and the results were expressed as mean ± S.D. Statistical analyses were performed using SPSS 19 (IBM, Armonk, NY, USA). One-way ANOVA followed by Tukey’s test was used for comparisons, with significance levels set at * p < 0.01 and ** p < 0.05.

## 3. Results

### 3.1. Identification and Sequence Characteristics of GCSF and MCSF in Starry Flounder

The ORF sequence of the starry flounder *GCSF* gene was 594 bp, encoding 198 amino acids (aa). It contained a specific IL-6 domain (33–185 aa), and this sequence information was registered in the NCBI database under the accession number PQ565685 (Table 2).

**Figure 1:**
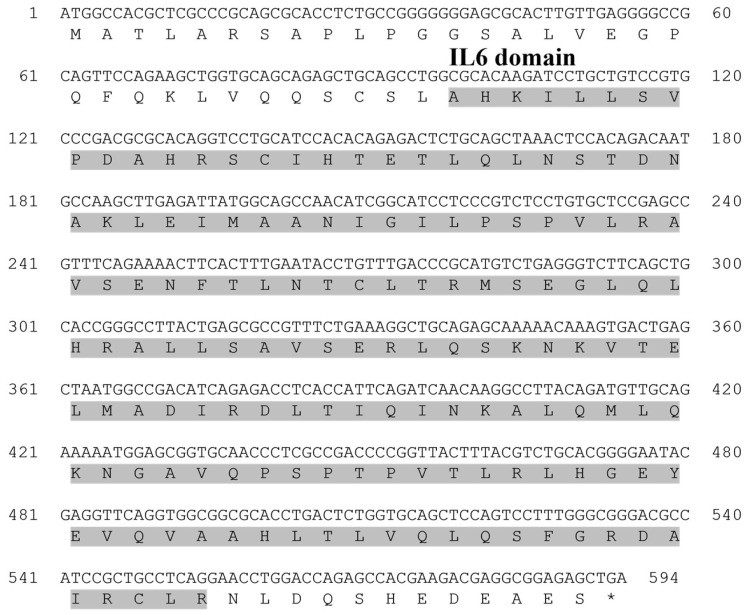
Nucleotide and deduced amino acid sequences of the granulocyte colony-stimulating factor (GCSF) from the starry flounder. The gray box indicates the interleukin-6 (IL-6) domain. The asterisk (*) indicates the stop codon in the amino acid sequence.

**Figure 2:**
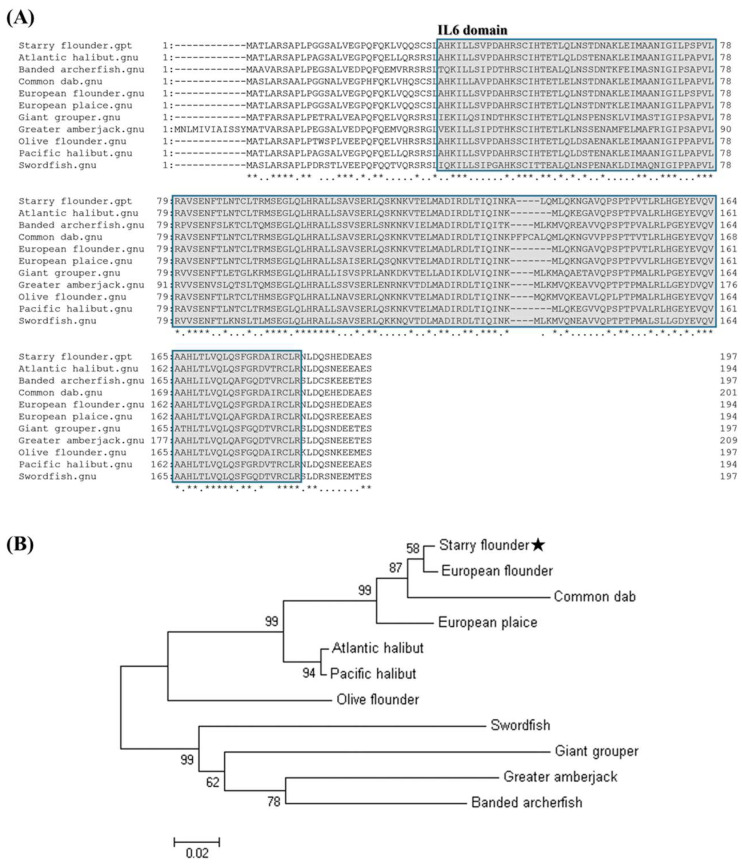
Multiple sequence alignment analysis of the deduced amino acid sequence of *GCSF* with other species’ *GCSF* sequences. The predicted IL-6 domain is indicated by gray boxes (**A**). Asterisks (*) indicate fully conserved residues among the aligned sequences. A phylogenetic tree of starry flounder *GCSF* and other known homologs based on the neighbor-joining (NJ) method. The scale bar indicates a branch length of 0.02. The numbers are bootstrap percentiles from 1000 replicates (**B**). The star (★) indicates the starry flounder used in this study.

The multiple sequence alignment of the amino acid sequence with the *GCSF* sequences reported in the other species revealed a high level of conservation across all the species (Figure 1), with sequence identities ranging from 72% to 97.97% (Table 2). The phylogenetic analysis showed that the starry flounder *GCSF* formed a distinct clade with the *GCSF* sequences from the other marine fish species. The closest phylogenetic relationship was observed with European flounder *GCSF* (Figure 2A,B).

The open reading frame (ORF) sequence of the starry flounder *MCSF* gene was 621 bp, encoding 207 amino acids (aa). It contained a transmembrane region (68–190 aa), and this sequence information was registered in the NCBI database under the accession number PQ565686 (Table 3).

**Figure 3:**
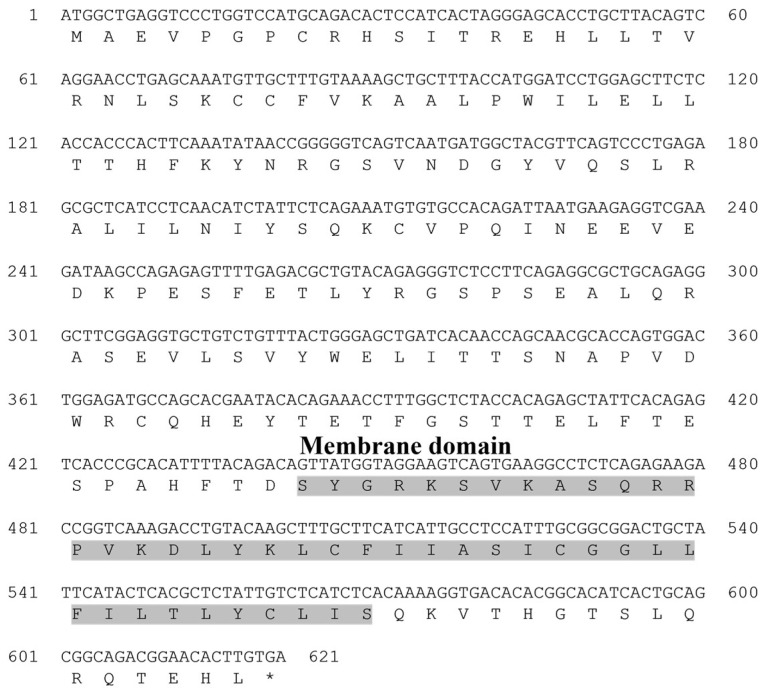
Nucleotide and deduced amino acid sequences of the macrophage colony-stimulating factor (MCSF) from the starry flounder. The gray box indicates the predicted transmembrane domain. The asterisk (*) indicates the stop codon in the amino acid sequence.

**Figure 4:**
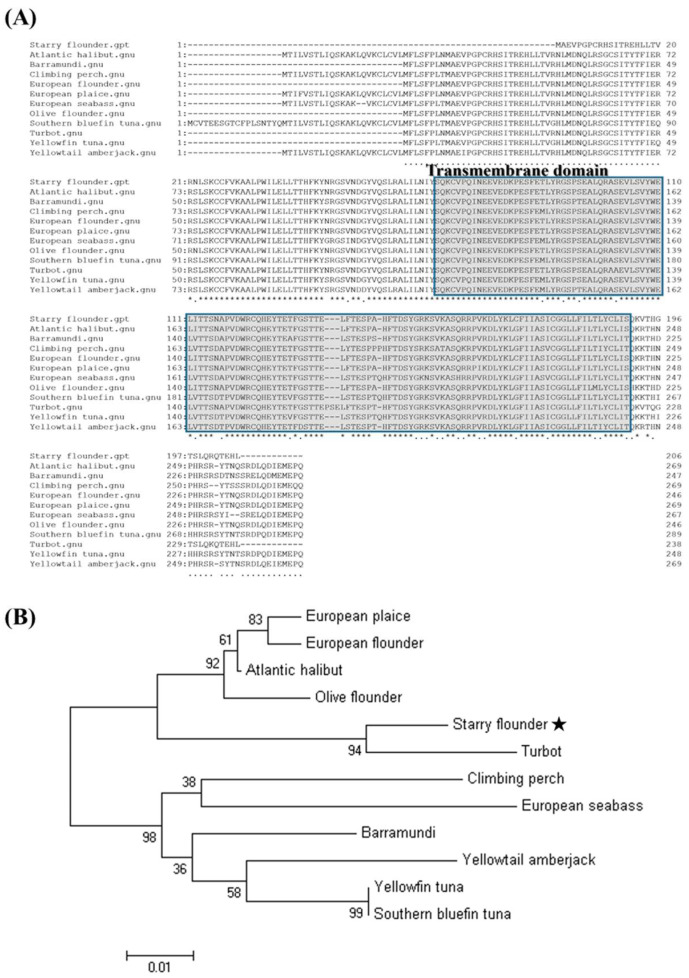
Multiple sequence alignment analysis of the deduced amino acid sequence of *MCSF* with other species *MCSF* sequences. The predicted transmembrane domain is indicated by gray boxes (**A**). Asterisks (*) indicate fully conserved residues among the aligned sequences. A phylogenetic tree of starry flounder *MCSF* and other known homologues based on the neighbor-joining (NJ) method. The scale bar indicates a branch length of 0.01. The numbers are bootstrap percentiles from 1000 replicates (**B**). The star (★) indicates the starry flounder used in this study.

The multiple sequence alignment of the amino acid sequence with the *MCSF* sequences reported in the other species revealed a high level of conservation across all the species (Figure 3), with sequence identities ranging from 82% to 86.28% (Table 3). The phylogenetic analysis showed that the starry flounder *MCSF* formed a distinct clade with the *MCSF* sequences from the other marine fish species. The closest phylogenetic relationship was observed with turbot *MCSF* (Figure 4A,B).

### 3.2. Expression Profiles of GCSF and MCSF in Starry Flounder

**Figure 5:**
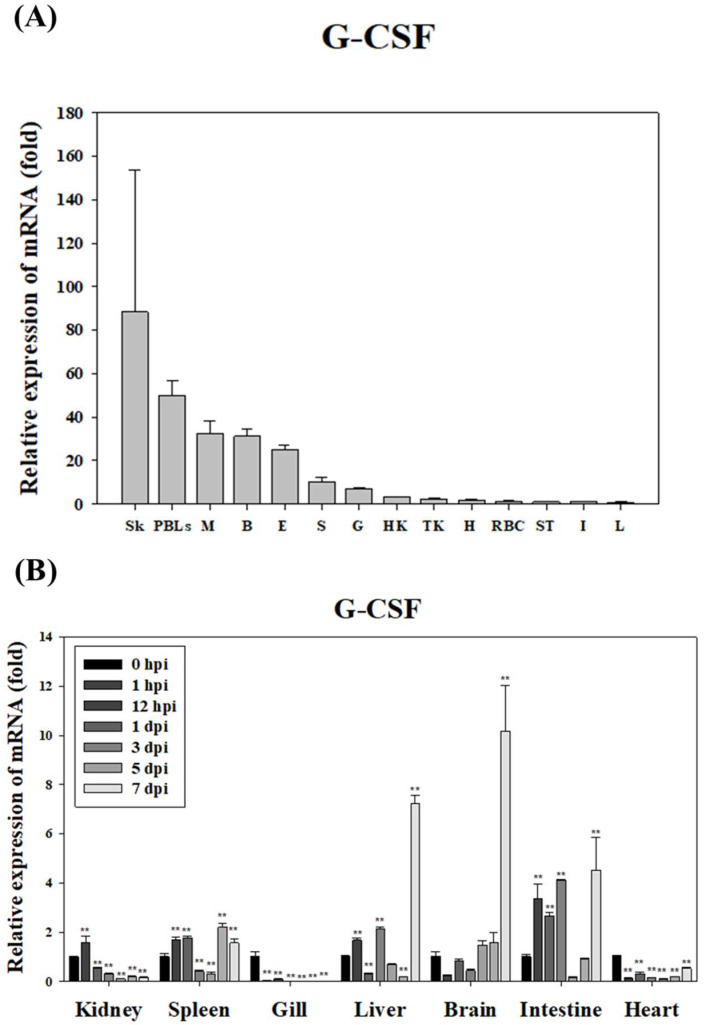
Expression level of *GCSF* mRNA in various tissues of the healthy-state starry flounder. The EF-1α gene was used to normalize the RT-qPCR results. The expression level is expressed as the fold change compared to the expression level of *GCSF* mRNA in the liver. All data are presented as the mean ± SD from five independent cDNA samples with three replicates per sample. Basal expression of GCSF mRNA in various tissues of the healthy starry flounder. Tissue abbreviations: Sk (skin), PBLs (peripheral blood leukocytes), M (muscle), B (brain), E (eye), S (spleen), G (gill), HK (head kidney), TK (trunk kidney), H (heart), RBC (red blood cell), ST (stomache), I (intestine), and L (liver) (**A**). The expression level of *GCSF* mRNA in the brain, gill, heart, head kidney, liver, intestine, and spleen of the starry flounder after infection with *S. parauberis*. Data represent mean ± SD (n = 5 fish per group). The gene expression levels and their significance are represented as the mean ± SD. Asterisks indicate significant differences (** *p* < 0.01) versus the control (0 h) (**B**). The 0 hpi group represents the samples collected immediately after bacterial injection and was used as an internal baseline control for assessing time-dependent gene expression in infected fish.

In healthy starry flounder, *GCSF* mRNA expression was lowest in the liver and highest in the skin, where it was expressed 88-fold higher. Elevated expression was also observed in the peripheral blood leukocytes (PBLs) and muscle, with expression levels 50-fold and 30-fold higher, respectively. However, relatively low expression was detected in the intestine, stomach, and red blood cells (RBCs) (Figure 5A). Following infection with *S. parauberis* PH0710, *GCSF* mRNA expression was significantly down-regulated in the gills and heart for most of the infection period. In contrast, significant up-regulation was observed in the brain, liver, and intestine, with the highest expression levels recorded on day 7 post-infection. Additionally, the kidney and spleen exhibited significant up-regulation of *GCSF* expression during the early stages of infection (Figure 5B).

**Figure 6:**
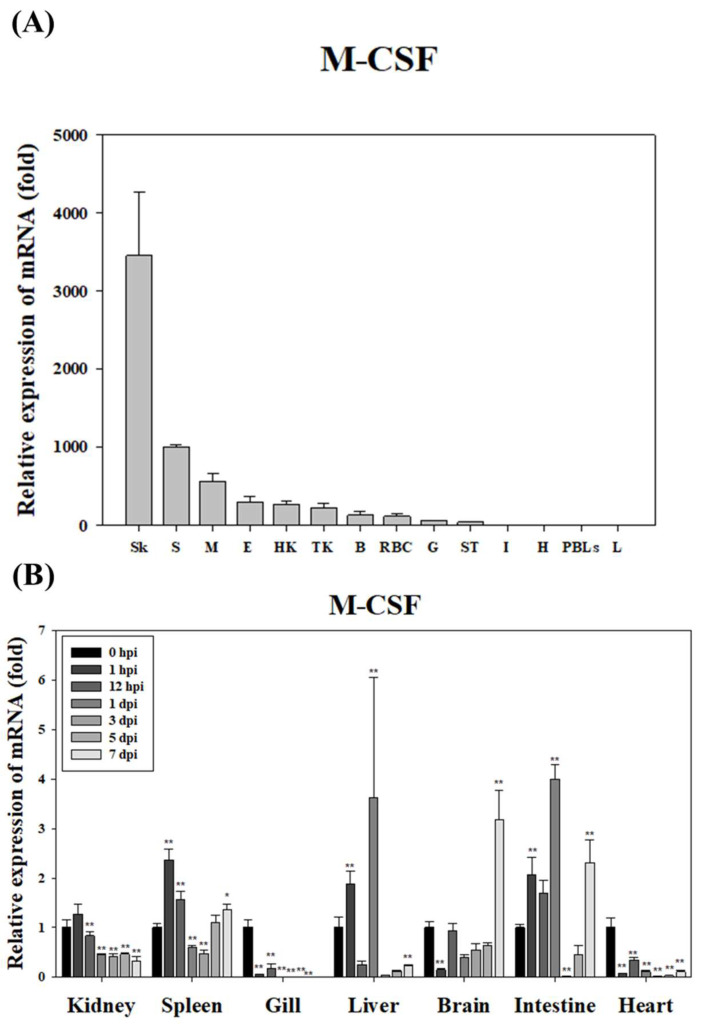
Expression level of *MCSF* mRNA in various tissues of the healthy state starry flounder. The EF-1α gene was used to normalize the RT-qPCR results. The expression level is expressed as the fold change compared to the expression level of *MCSF* mRNA in the liver. All data are presented as the mean ± SD from five independent cDNA samples with three replicates per sample. Basal expression of GCSF mRNA in various tissues of the healthy starry flounder. Tissue abbreviations: Sk (skin), S (spleen), M (muscle), E (eye), HK (head kidney), TK (trunk kidney), B (brain), RBC (red blood cell), G (gill), ST (stomache), I (intestine), H (heart), PBLs (peripheral blood leukocytes), and L (liver) (**A**). The expression level of *MCSF* mRNA in the brain, gill, heart, head kidney, liver, intestine, and spleen of the starry flounder after infection with *S. parauberis*. Data represent mean ± SD (n = 5 fish per group). The gene expression levels and their significance are represented as the mean ± SD. Asterisks indicate significant differences (* *p* < 0.05 and ** *p* < 0.01) versus the control (0 h) (**B**). The 0 hpi group represents the samples collected immediately after bacterial injection and was used as an internal baseline control for assessing time-dependent gene expression in infected fish.

In the healthy starry flounder, *MCSF* mRNA expression was lowest in the liver and highest in the skin, where it was expressed 3454-fold higher. High expression levels were also observed in the spleen (1008-fold) and muscle (563-fold), while lower levels were found in PBLs, the heart, and the intestine (Figure 6A). During infection with *S. parauberis* PH0710, *MCSF* mRNA expression was significantly down-regulated in the gills and heart throughout most of the infection period. However, except for the gills and heart, significant up-regulation was detected in the majority of tissues during the early stages of infection (Figure 6B).

### 3.3. Production of Recombinant GCSF and MCSF Protein

**Figure 7:**
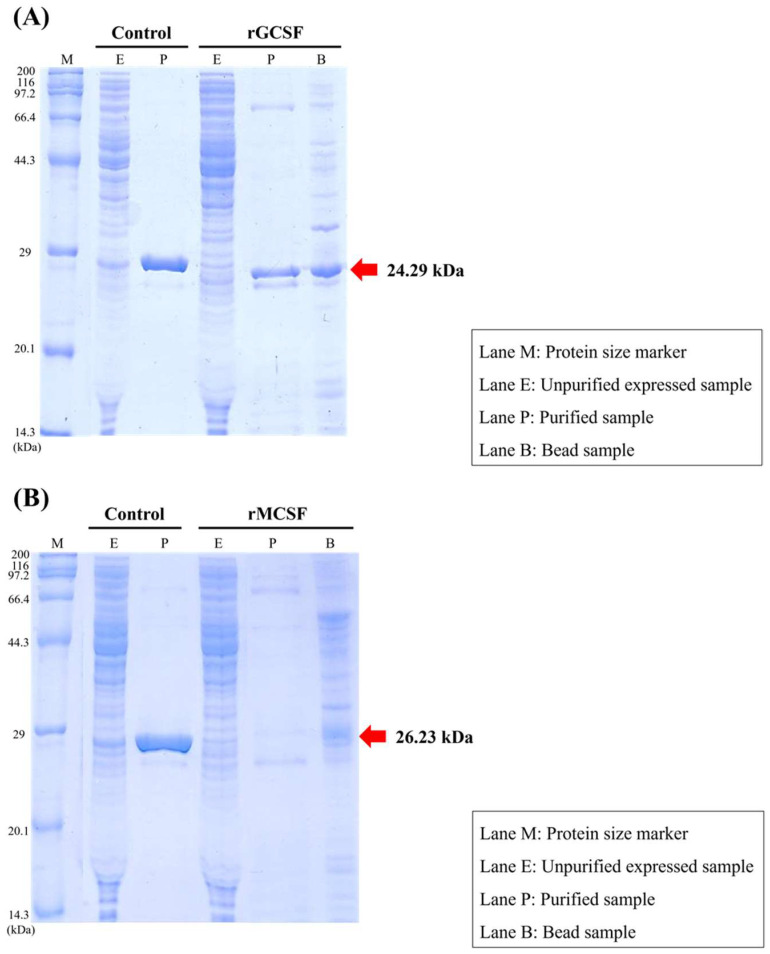
The 12% SDS-PAGE analysis of *GCSF* recombinant protein. Control: AcGFP is a basic (constitutively fluorescent) green fluorescent protein, derived from *Aequorea coerulescens*. Lane M: molecular weight marker; Lane E: unpurified expressed sample; Lane P: purified sample; Lane B: bead sample (**A**). 12% SDS-PAGE analysis of *MCSF* recombinant protein. Control: AcGFP is a basic (constitutively fluorescent) green fluorescent protein, derived from *Aequorea coerulescens*. Lane M: molecular weight marker; Lane E: unpurified expressed sample; Lane P: purified sample; Lane B: bead sample (**B**). The expected molecular weight of *rMCSF* with the 6×His-tag was ~26.2 kDa. Although expression was confirmed by SDS-PAGE, the target protein was not successfully purified, possibly due to improper folding or aggregation.

The molecular weight of *GCSF* was predicted to be 21.55 kDa, and including the 6×His-tag fusion partner (~2.7 kDa), the total molecular weight of the recombinant protein was predicted to be 24.29 kDa (Figure 7A). Following the production of *rGCSF*, its molecular weight was confirmed via 12% SDS-PAGE, showing the successful production of the specific target protein.

The molecular weight of *MCSF* was predicted to be 23.49 kDa, and including the 6×His-tag fusion partner (~2.7 kDa), the total molecular weight was predicted to be 26.23 kDa (Figure 7B). Although the 12% SDS-PAGE analysis confirmed the expression of *rMCSF*, purification using Ni-affinity chromatography was unsuccessful, likely due to misfolding or the formation of inclusion bodies. The detected band was smaller than the expected ~26.2 kDa, and the protein could not be recovered in a soluble, purified form.

### 3.4. Phagocytic Activity of Recombinant GCSF Protein

The ability of *rGCSF* to induce phagocytic activity in the peripheral blood leukocytes (PBLs) and trunk kidney-derived lymphocytes (TK-derived PBLs) of the starry flounder was evaluated. A gating analysis was performed to identify cell populations in the PBLs and TK-derived PBLs, and the proportion of phagocytic cells after *rGCSF* stimulation was assessed.

**Figure 8:**
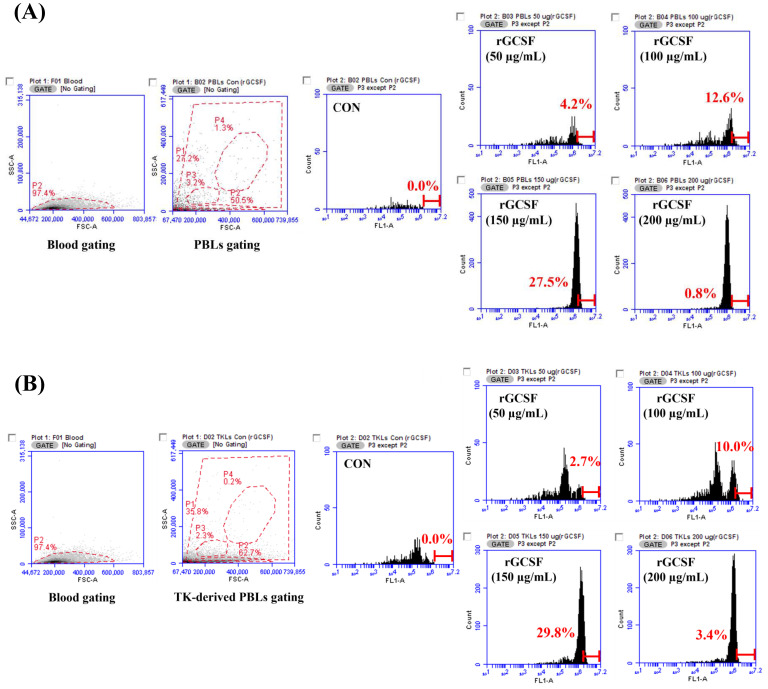
Evaluation of phagocytic activity in PBLs following treatment with *rGCSF*. Flow cytometry analysis of the peripheral blood leukocytes (PBLs) from the starry flounder treated with varying concentrations of *rGCSF*. The PBLs were gated for analysis, as shown in the left panels. (**A**) Evaluation of phagocytic activity in the TK-derived PBLs following treatment with *rGCSF*. Flow cytometry analysis of the kidney-derived peripheral blood leukocytes (TK-derived PBLs) from the starry flounder treated with various concentrations of *rGCSF*. Gating for the TK-derived PBLs is shown in the left panels (**B**). The dashed red line represents the gating region used to identify peripheral blood leukocytes (PBLs) after excluding debris (P1). Solid red lines indicate gated subpopulations: P2 represents erythrocytes, P3 indicates monocytes, and P4 represents granulocytes.

In the PBLs, phagocytic activity increased from 4.2% at 50 μg/mL to 12.6% at 100 μg/mL, and peaked at 27.5% at 150 μg/mL, compared to the negative control (CON). However, at 200 μg/mL, the phagocytic activity decreased to 0.8% (Figure 8A).

Similarly, in the TK-derived PBLs, phagocytic activity increased from 2.7% at 50 μg/mL to 10.0% at 100 μg/mL, and reached its highest value at 29.8% at 150 μg/mL. At 200 μg/mL, the phagocytic activity decreased to 3.4% (Figure 8B).

### 3.5. Cytotoxicity of the Recombinant GCSF Protein

**Figure 9:**
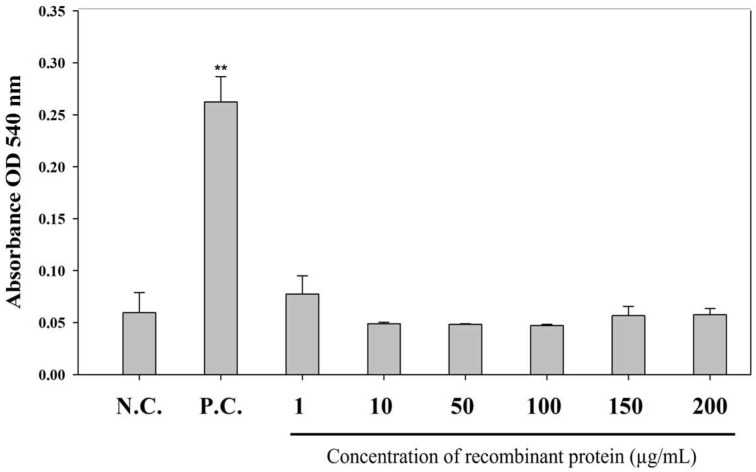
The haemolytic and cytotoxicity of *rGCSF* analyzed using the starry flounder red blood cells. The positive control was measured by adding 0.1% Triton X-100 solution. Values are expressed as the mean ± SD, and asterisks indicate a significant difference (** *p* < 0.01) from the negative control (PBS buffer).

To evaluate the in vivo safety of *rGCSF*, hemolytic activity was measured in the starry flounder erythrocytes. Unlike the positive control, no hemolysis was observed in the *rGCSF*-treated groups, even at the highest concentration of 200 μg/mL, which was comparable to the PBS buffer-treated control group (Figure 9).

## 4. Discussion

In this study, *GCSF* and *MCSF* were identified in the starry flounder through NGS analysis, and their sequence characteristics were analyzed. Specific domains and motifs of these genes were identified using the PROSITE profile database and the Simple Modular Architecture Research Tool. Multiple sequence alignments and phylogenetic analyses were performed to determine the potential roles of these genes in the immune response of starry flounder.

*GCSF* is a key cytokine that regulates the proliferation, survival, and differentiation of various immune cells, including neutrophils, monocytes, macrophages, and their precursors [10]. It plays a broad role in antimicrobial immune responses. In this study, the *PsGCSF* gene was identified as being 594 bp long, encoding a sequence of 198 amino acids. Structural analysis revealed that the protein possesses a conserved *GCSF* structure, including a characteristic IL-6 domain. This conserved structure, a hallmark of the *GCSF* family, is known to mediate acute-phase responses, such as microbial infections. Multiple sequence alignment showed high sequence conservation among species, with *PsGCSF* sharing 97.97% sequence similarity with the *GCSF* of the European flounder. The phylogenetic analysis clustered *PsGCSF* closely with the *GCSF* from other marine fish, indicating its evolutionary conservation. These findings suggest that *PsGCSF* plays a critical role in immune responses, functioning as a mediator of acute-phase reactions and host defense mechanisms in fish.

*MCSF* promotes the proliferation of macrophages, which play a critical role in phagocytosing and eliminating pathogens in infected tissues [21]. The *PsMCSF* gene identified in this study is 621 bp long, encoding a sequence of 207 amino acids. The structural analysis confirmed the presence of a conserved *MCSF* protein structure, including a transmembrane region. The multiple sequence alignment revealed high sequence conservation across species, with *PsMCSF* sharing 86.28% similarity with the *MCSF* of turbot. The phylogenetic analysis grouped *PsMCSF* with the *MCSF* from other marine fish, highlighting its evolutionary conservation. These results confirm the functional significance of *PsMCSF* as an immune-related gene involved in pathogen clearance and immune regulation.

Changes in mRNA expression are sensitive indicators of biological activity. RNA-based expression studies, which began with the development of Northern blotting by [22], have since evolved with the adoption of diverse analytical methods. In this study, the tissue-specific mRNA expression profiles of the starry flounder were analyzed to understand the functions and regulatory mechanisms of the identified immune-related genes.

The expression characteristics of *GCSF* in the starry flounder suggest its critical role in host immune defense against various pathogens. *GCSF* is known to play an essential role in regulating the proliferation and differentiation of immune cells [8,10], as well as the survival and function of key immune cells such as neutrophils and macrophages. According to previous studies, *GCSF* expression in mammals, including humans, significantly increases in response to pathogen infection and inflammatory stimuli, serving as a vital mechanism for enhancing host immune responses.

A similar pattern has been observed in fish. In black rockfish, *GCSF*-1 expression was induced in response to various stimuli such as LPS, Con A/PMA, and Poly I:C [23]. Additionally, in rockfish, pathogens like *Streptococcus iniae* were shown to increase *GCSF* expression in the kidney, spleen, and gills [24]. These induced responses varied across tissues depending on the type of pathogen, indicating that *GCSF* expression is regulated by interactions between the pathogen’s characteristics and the host tissue.

In the case of starry flounder, *PsGCSF* expression was found to be induced in specific tissues following bacterial pathogen infection with *S. parauberis*. Notable increases in *PsGCSF* expression were observed in immune-related tissues such as the head kidney, spleen, and liver, suggesting that *GCSF* plays a crucial role in the early stages of immune cell recognition and defense against pathogens. This indicates that *PsGCSF* may act as a key mediator in regulating the innate immune response of starry flounder and responding to pathogen infections.

Moreover, the inducible expression of *PsGCSF* in response to specific pathogens highlights its potential significance in mediating interactions with various pathogens. Previous studies in olive flounder provide additional context, showing that *GCSF* expression patterns differ depending on the pathogen, such as *Vibrio anguillarum*, *Edwardsiella tarda*, and ISKNV [25]. These findings suggest that *PsGCSF* might perform distinct roles in pathogen-specific immune responses and possess the capability to finely regulate tissue-specific responses as part of defense mechanisms.

*MCSF* is a key cytokine involved in the survival and differentiation of macrophages, playing an important role in immune responses in mammals [6]. In this study, high expression of *MCSF* was observed in the skin, spleen, and muscle of the healthy starry flounder, suggesting that macrophages are actively present at pathogen-contact sites, where they perform immune defense functions. Following infection, *MCSF* expression was down-regulated in the gills and heart but significantly up-regulated in other tissues during the early stages of infection. This indicates a role of macrophage activation and the tissue-specific regulation of immune responses in response to infection [26], suggesting that *MCSF* plays a critical role in pathogen elimination in fish. These findings are consistent with the study by [27], which reported the importance of macrophage activation in the early immune response in fish.

Recombinant proteins of the key immune-related genes in the starry flounder *GCSF* and *MCSF* were produced, and their expression and purification were evaluated. While *GCSF* was successfully purified, *MCSF* showed incomplete folding during structural formation, leading to the formation of inclusion bodies. As a result, optimal outcomes were not achieved during purification. Future studies should focus on improving purification methods to ensure the proper structural formation of these proteins.

To confirm the potential of the produced *rGCSF* as a molecular adjuvant, a series of functional assays were conducted to assess its ability to enhance phagocytic activity and cytotoxicity. The recombinant proteins *rGCSFs* demonstrated their potential roles in enhancing immune responses through phagocytic activity and cytotoxicity assays. For *rGCSF*, phagocytic activity in PBLs increased with higher concentrations, reaching its peak at 150 μg/mL with a phagocytic activity of 27.5%. However, at 200 μg/mL, the phagocytic activity decreased, suggesting that excessive cellular stimulation at high concentrations may have adverse effects, underscoring the importance of determining the optimal concentration. Similar patterns were observed in the TK-derived PBLs, where the maximum phagocytic activity was observed at 150 μg/mL, followed by a decline at 200 μg/mL. Previous studies in mammals have shown that *rGCSF* enhances immune responses to pathogen infection by increasing the number and functionality of neutrophils [28]. Similarly, in starry flounder, *rGCSF* appears to enhance host resistance to pathogen infections. This study demonstrated the role of *PsGCSF* in enhancing host immune responses through phagocytic activity, providing important insights into the function of GCSF in marine fish like starry flounder.

Additionally, to evaluate the safety of *rGCSF*, hemolytic activity was assessed using the red blood cells from the starry flounder. The results showed no hemolysis compared to the PBS-treated control group, confirming the absence of cytotoxicity. These findings suggest that *rGCSF* can be safely utilized within the body of starry flounder.

Previous studies have also shown that the effects of immune adjuvants are not necessarily dose-proportional, and higher doses may suppress immune responses. For instance, injections of levamisole at 0.1 and 0.5 mg/kg enhanced phagocytic activity in rainbow trout, but this effect was not observed at a dose of 5 mg/kg [29]. Similarly, previous studies demonstrated that respiratory burst activity in macrophages treated with glucan peaked at concentrations of 0.1–1 μg/mL, showed no effect at 10 μg/mL, and was inhibited at 50 μg/mL [30]. Consistent with these findings, this study observed a decrease in phagocytic activity at 200 μg/mL when *rGCSF* was applied to the starry flounder, underscoring the need for optimal dose determination.

In summary, our study underscores the critical roles of *GCSF* and *MCSF* in mediating the immune responses of starry flounder, particularly in the context of pathogen infection by *S. parauberis*. The structural and phylogenetic analyses confirmed the evolutionary conservation of these cytokines, while the expression profiling revealed their tissue-specific regulation and inducibility upon infection. The functional assays demonstrated that recombinant *GCSF* effectively enhances phagocytic activity and maintains cellular safety, suggesting its promising potential as a molecular adjuvant. However, the challenges encountered in achieving the proper folding and purification of *MCSF* highlight the need for further optimization. These findings not only provide valuable insights into the molecular mechanisms underlying host defense in marine fish but also pave the way for future research aimed at developing effective molecular adjuvants to improve disease resistance and productivity in aquaculture.

## 5. Conclusions

In this study, we have delineated the molecular characteristics and functional potential of two key cytokines, *GCSF* and *MCSF*, in starry flounder. Both genes exhibit conserved structural motifs and phylogenetic relationships consistent with other marine teleosts, and their expression is markedly induced in barrier tissues and primary immune organs following bacterial challenge. Recombinant *GCSF* produced via a cell-free system significantly promotes leukocyte phagocytic activity at 150 µg/mL without causing hemolytic toxicity, underscoring its promise as a safe and effective molecular adjuvant in aquaculture.

Conversely, challenges in the proper folding and purification of *rMCSF* highlight the necessity for further optimization of expression and refolding protocols. Future work should, therefore, focus on in vivo vaccination trials combining *rGCSF* with model antigens, refinement of *rMCSF* production methods, and exploration of synergistic effects between multiple cytokine adjuvants. Collectively, these efforts will contribute to the development of novel immunostimulatory strategies aimed at enhancing disease resistance and productivity in high-value aquaculture species.

## Figures and Tables

**Table 1:** List of primer sets used for RT-qPCR.

Usage	Primer	Primer Sequence (5′-3′)	Efficiency (%)	R^2^	Pearson’s r
Housekeeping gene	EF-1α (F)	GTGGCAAGTCCACCACCA	97.2	0.998	0.998
	EF-1α (R)	GCTTGTCCAGCACCCAGG			
Target gene	GCSF (F)	CTTACTGAGCGCCGTTTCTG	94.5	0.997	0.996
	GCSF (R)	TGGTGAGGTCTCTGATGTCG			
	MCSF (F)	AGGAAGTCAGTGAAGGCCTC	92.8	0.996	0.995
	MCSF (R)	CCGCAAATGGAGGCAATGAT			

F = forward primer; R = reverse primer; Efficiency (%) = qPCR amplification efficiency; R^2^ = coefficient of determination; Pearson’s r = Pearson correlation coefficient.

**Table 2:** Sequence homology (%) of *GCSF* between starry flounder and other species.

Species	Domain Length (aa)	GenBank Accession Number	Sequence Homology
*Starry flounder*	153	PQ565685	-
*European flounder*	150	XP_062234477.1	97.97%
*European plaice*	150	XP_053270026.1	94.42%
*Common dab*	157	XP_060951581.1	91.54%
*Atlantic halibut*	153	XP_034426689.1	91.37%
*Pacific halibut*	150	XP_035001744.1	90.86%
*Olive flounder*	153	XP_019938527.1	81.73%
*Banded archerifish*	153	XP_040922992.1	75.13%
*Swordfish*	153	XP_039991573.1	73.60%
*Giant grouper*	153	XP_033466924.1	72.08%

**Table 3:** Sequence homology (%) of *MCSF* between starry flounder and other species.

Species	Domain Length (aa)	GenBank Accession Number	Sequence Homology
*Starry flounder*	23	PQ565686	-
*European plaice*	23	XP_053281761.1	86.28%
*European flounder*	23	XP_062247107.1	86.28%
*Atlantic halibut*	23	XP_034446716.1	86.28%
*Turbot*	23	AWP09878.1	86.52%
*Olive flounder*	23	XP_019951579.1	85.40%
*Barramundi*	23	XP_018541570.1	84.72%
*Yellowfin tuna*	23	XP_044206290.1	83.87%
*Southern bluefin tuna*	23	XP_042265693.1	83.87%
*European seabass*	23	XP_051280811.1	82.11%
*Climbing perch*	23	XP_026222830.1	82.11%
*Yellowtail amberjack*	23	XP_023278526.1	82.03%

## Data Availability

The datasets used and/or analyzed during the current study are available from the corresponding author on reasonable request.
